# Supplementary material for: Pretreatment Proteinuria Predicts the Prognosis of Patients Receiving Systemic Therapy for Unresectable Hepatocellular Carcinoma
Source: Cancers (Basel). 2023 May 21;15(10):2853. doi: 10.3390/cancers15102853 (PMC10216745; doi:10.3390/cancers15102853)
Supplement: Supplementary file 1 [file cancers-15-02853-s001.zip › cancers-2334512-supplementary.pdf]

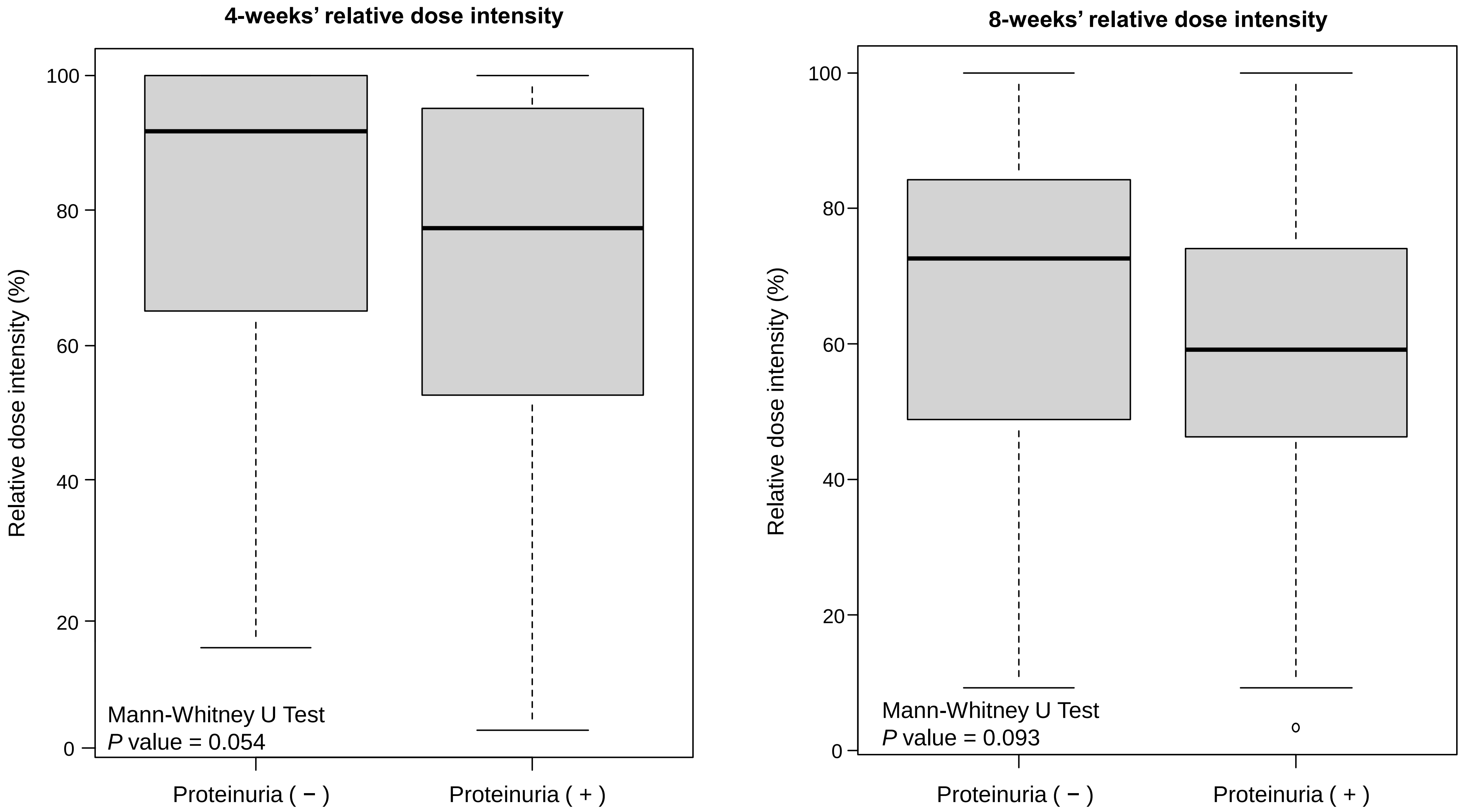

Supplemental Figure S1. Relative dose intensity of lenvatinib based on pretreatment proteinuria.
Positive pretreatment proteinuria was defined as a ratio of urinary protein to creatinine of ≥ 0.15 g/g creatinine. Within each box, horizontal black lines denote median values; boxes extend from the 25th to the 75th percentile of each group's distribution of values; vertical extending lines denote adjacent values (i.e., the most extreme values within 1.5 interquartile range of the 25th and 75th percentile of each group); dots denote observations outside the range of adjacent values.

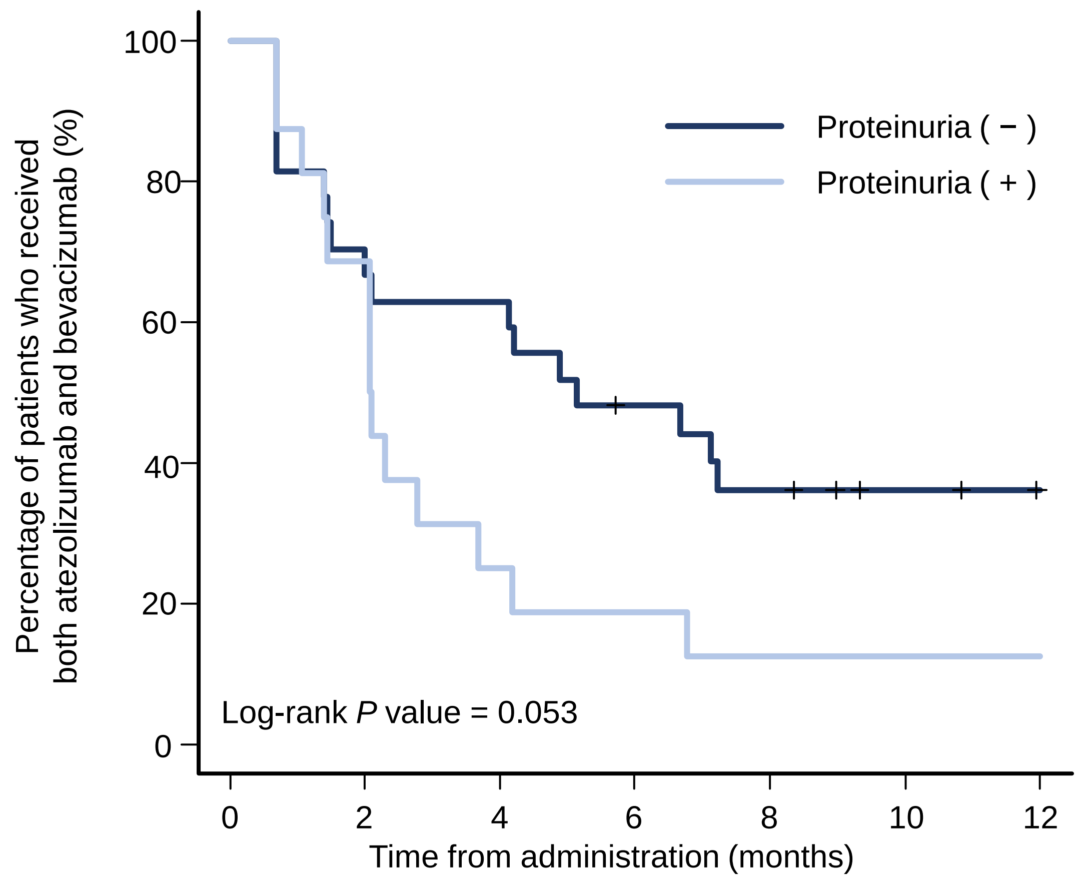

Number at risk							
Proteinuria (−)	27	19	17	12	9	6	4
Proteinuria (+)	16	11	4	3	2	2	2

Supplemental Figure S2. Kaplan-Meier analysis of treatment duration with atezolizumab and bevacizumab without discontinuation of bevacizumab due to adverse events.
Kaplan-Meier estimates were calculated for patients who received atezolizumab and bevacizumab without skipping bevacizumab doses due to adverse events. Positive pretreatment proteinuria was defined as a ratio of urinary protein to creatinine of ≥ 0.15 g/g creatinine.
